# The New Perspectives on Genetic Studies of Type 2 Diabetes and Thyroid Diseases

**DOI:** 10.2174/138920213804999138

**Published:** 2013-03

**Authors:** Min Xu, Yufang Bi, Bin Cui, Jie Hong, Weiqing Wang, Guang Ning

**Affiliations:** 1 Key Laboratory for Endocrine and Metabolic Diseases of Ministry of Health, Rui-Jin Hospital, Shanghai Jiao Tong University School of Medicine, E-Institute of Shanghai Universities, Shanghai, China; 2 Shanghai Clinical Center for Endocrine and Metabolic Diseases, Shanghai Institute of Endocrine and Metabolic Diseases, Department of Endocrinology and Metabolism, Rui-Jin Hospital, Shanghai Jiao Tong University School of Medicine, Shanghai, China

**Keywords:** Genome wide association study, Gene-environmental interaction, Hyperthyroidism, Risk prediction, Type 2 diabetes.

## Abstract

Recently, genome-wide association studies (GWAS) have led to the discovery of hundreds of susceptibility loci that are associated with complex metabolic diseases, such as type 2 diabetes and hyperthyroidism. The majority of the susceptibility loci are common across different races or populations; while some of them show ethnicity-specific distribution. Though the abundant novel susceptibility loci identified by GWAS have provided insight into biology through the discovery of new genes or pathways that were previously not known, most of them are in introns and the associated variants cumulatively explain only a small fraction of total heritability. Here we reviewed the genetic studies on the metabolic disorders, mainly type 2 diabetes and hyperthyroidism, including candidate genes-based findings and more recently the GWAS discovery; we also included the clinical relevance of these novel loci and the gene-environmental interactions. Finally, we discussed the future direction about the genetic study on the exploring of the pathogenesis of the metabolic diseases.

## INTRODUCTION

The genetic research of complex diseases has achieved remarkable leap during the past several years since the completion of the first genome-wide association study (GWAS) of age-related macular degeneration has been published in 2006 [1]. Such revolutionary progress in the field is largely due to the breakthrough in genotyping technology. GWAS has been extensively employed in genetic analysis of various human diseases (e.g., diabetes, obesity, cancers, cardiovascular diseases, dyslipidemia, neuropsychiatric diseases, autoimmune diseases, and infectious diseases) as well as disease-related quantitative traits (e.g., body height, blood glucose levels, body mass index (BMI) and waist circumference). GWAS has led to the discovery of hundreds of susceptibility loci that are associated with complex endocrine and metabolic traits, as long as diseases, such as type 2 diabetes (T2D), obesity and hyperthyroidism, and so on.

The metabolic diseases rose rapidly in the past decades and the number of adults with diabetes is expected to rise to about 440 million by 2030 almost 80% of whom will be from low-income and middle-income countries [2, 3]. T2D is a chronic complex metabolic disorder, the pathogenesis of which is not well elucidated though the impaired insulin sensitivity and islet ( cell dysfunction being the two main mechanisms. Besides the environmental or lifestyle risk factors, like age, obesity, excess energy and longer sedentary time, etc, the genetic risk factors play a pivotal role in the incidence of T2D.

Hyperthyroidism is a condition in which the thyroid gland makes too much thyroid hormone, and the most common causes of hyperthyroidism are Graves' disease, followed by toxic multinodular goitre, whilst rarer causes include an autonomously functioning thyroid adenoma, or thyroiditis [4]. Hyperthyroidism is often referred to as an "overactive thyroid." Hyperthyroidism occurs when the thyroid releases too much of its hormones over a short (acute) or long (chronic) period of time. Many diseases and conditions can cause this problem, including: Graves disease (accounts for most cases of hyperthyroidism), inflammation (thyroiditis) of the thyroid due to viral infections or other causes, noncancerous growths of the thyroid gland or pituitary gland [5]. Graves’ disease is a common organ-specific autoimmune disease, which is, to a significant extent, determined by genetic factors [6, 7]. The search for gene variations that predispose to such disease is complicated by their polygenic nature. 

## THE CANDIDATE GENE ASSOCIATION STUDIES OF T2D

Before the GWAS era, linkage analysis and candidate genes analysis are the two main methods to explore the effect of genetic factors on T2D. The unequivocal established susceptible loci for the common type of T2D have limited to CAPN10, TCF7L2, KCNJ11 and PPARG genes. CAPN10 and TCF7L2 are the two genes successfully identified by the linkage analysis. CAPN10, which encodes the cysteine protease calpain 10, was the first T2D susceptibility gene identified through a genome-wide linkage followed by positional cloning [8]. Many validated studies have been performed from Caucasians to East Asians [9-1]. TCF7L2, which encodes the transcription factor 7-like 2, was firstly found to be associated with T2D in Danish and US cohorts, through fine-mapping of a suggestive linkage to chromosome 10 [12]. After that, this gene was extensively and successfully replicated and validated in many populations, including the Indians, French and Asians, etc [13-17]. KCNJ11 and PPARG are the two proven susceptibility genes for T2D that was confirmed by candidate gene methods [18, 19]. The mostly studied polymorphisms associated with T2D are E23K in KCNJ11 and P12A in PPARG. KCNJ11, namely potassium inwardly-rectifying channel, subfamily J, member 11, encodes inward rectifier K (+) channel Kir6.2 (KIR6.2), which is important on the effect of anti-diabetic drug sulphonylureas. PPARG encodes peroxisome proliferator-activated receptor gamma, which is a target of thiazolidinediones. PPARG gene is one of the well-established susceptible genes of T2D. Interestingly, PPARG is one of the few genes that were confirmed to be associated with insulin resistance, Significantly greater insulin sensitivity was reported in not only nondiabetic alanine (Ala) carriers, but also the diabetic patients [20, 21].

## NEW SUSCEPTIBILITY GENES WERE IDENTIFIED BY GWAS

Since the first GWAS, the number of susceptibility loci for T2D has grown up to more than 50 (Table **1**) [22-45]. Most of the susceptibility loci are successfully validated in different races or ethnic groups. However, there are ethnicity-specific genetic loci have also been identified. Rs7903146 of TCF7L2 was widely accepted as one of the most relative susceptibility single nucleotide polymorphism (SNP) with T2D, which was replicated in almost all the GWAS [22, 24, 28, 29, 31, 34, 35]. However, they were mostly performed in Caucasians, and much less GWAS was conducted in Asian populations [27, 33, 37-39, 41, 46]. The minor allele frequency (MAF) of this variation may make the difference. The MAF of rs7903146 in the TCF7L2 gene in East Asians is 0.024–0.042 in control subjects and 0.023–0.055 in patients with T2D [17, 47-49]. In Caucasians, the MAF is 0.180–0.305 in control subjects and 0.220–0.425 in patients with T2D [12-16]. The less frequency of the polymorphisms may lead to less power to be detected in the association study. 

Another discrepancy lies in KCNQ1 gene. KCNQ1 was thought to be an Asian-specific susceptibility gene for T2D when it was firstly detected by GWAS in Japanese [27, 33] and followed by multiple replication studies in other Asian populations [30, 38, 46]. The previously reported GWAS performed in Europeans and Caucasians did not identify KCNQ1 until the large-scale combining genome-wide association data from European descent reported a second independent signal of KCNQ1, rs231362 [50], which is different from the previously reported ones among Asian populations (rs2237892[33], rs2237895[38], rs2237897[27], rs163182 [46]). The MAF of rs231362 in Caucasians is 0.52, which is much higher than 0.08 for rs2237892 and 0.05 for rs2237897.

The remarkable findings from GWAS have inspired investigators and the medical professionals to think about the clinical utility and the impact of their results. One of these considerations is whether it could be effective to discover the functional variations, the ‘causal’ variants. Though GWAS is a powerful way to rapidly and systematically identify new associations, it cannot refine a direct association between a disease or trait and the “causal” DNA sequences (causal in the sense that altering these sequences would eliminate the diabetic phenotype). To the date, the role of GWAS loci in T2D development is less established. With few exceptions such as KCNJ11 and SLC30A8 whose functions are well studied, the causal variant(s), causal gene(s) and pathophysiological processes implicated in GWAS loci (independently and in combination) are little understood. 

However, the present GWAS and primary functional studies have achieved some progression on genes in cell cycling control (CDKN2A/2B, CDKAL1), transcription factors (TCF7L2, HHEX), and ion channels (SLC30A8, KCNQ1). Two common variants (near or in FTO and MC4R) alter diabetes risk mediated by a primary effect of obesity [51]. There are many epidemiologic or *in vivo* function studies which have shown that most of the genetic loci of T2D are associated with the islet ( cell function. The genes identified by GWAS are mostly involved in the process of insulin synthesis and secretion, and seldom are in the process of insulin effect on the target organs. This has been viewed as presumptive evidence that insulin secretion plays a more important etiologic role in T2D than insulin resistance. TCF7L2 is the mostly explored susceptible gene for T2D. Common SNPs in TCF7L2 are reproducibly associated with T2D and reduced insulin response to glucose in nondiabetic individuals [52-54]. Lyssenko and his colleagues extensively explored the predictive effect of 3 SNPs (rs7903146, rs12255372, and rs10885406) in TCF7L2 and the mechanisms in Scandinavians, Swedish and Finnish. They concluded that the increased risk of T2D conferred by variants in TCF7L2 involves the enteroinsular axis, enhanced expression of the gene in islets, and impaired insulin secretion [55]. The common variations of SLC30A8 also have also been extensively studied in a great deal of populations [24, 28, 32, 56, 57]. SLC30A8 encoded the zinc transporter 8 (ZnT8), a member of the zinc transporter (ZnT/Slc30) family) [58, 59]. Both *in vitro* systems and *in vivo* studies in the knockout mice and humans, [60-63] have implicated ZnT8 in the development of T2D and are closely related to insulin synthesis and/or secretion. Another extensively studied susceptibility gene is KCNQ1, which was also reported to be highly related to β cell function [64, 65]. Many of the T2D susceptibility genes identified by GWAS affect β cell function (cell cycle regulation), and only a limited number of T2D GWAS loci are associated with insulin resistance (e.g., PPARG, FTO, IRS1 and KLF14) [34]. On one hand, these findings highlight the significant role of β cell dysfunction in T2D pathogenesis; on the other hand, the environmental impact on the development of insulin resistance and case-control design render it much more difficult to identify genetic loci associated with insulin resistance than those with β cell function [66]. Insulin resistance and obesity are highly correlated, and thus by deliberately minimizing the confounding influence of obesity, those scans maximized the chances of identifying insulin secretion genes. One example is that the Welcome Trust Case Control Consortium (WTCCC) identified a locus near FTO associated with T2D in analysis without adjustment for BMI. When the BMI effect was statistically accounted for the association disappeared, indicating that the diabetes risk associated with the FTO locus is mediated by obesity [67]. Insulin resistance genes may also have smaller effect sizes which the current GWAS were underpowered to detect, may be relatively rare and not tagged by the current set of SNPs, or their manifestation may be subjected to stronger environmental influences [66].

## CLINICAL CORRELATION OF T2D SUSCEPTIBILITY LOCI IDENTIFIED BY GWAS

Clinical application of T2D GWAS loci is limited mainly due to the lack of information regarding biological function, the small proportion of the heritability explained by the common variants and the minor discrimination effect added to the conventional clinical factors.

Though the abundant novel susceptibility loci identified by GWAS have provided insight into biology through the discovery of new genes or pathways that were previously unknown, most of them are in introns, showing a moderate effect (Table **1**) and the associated variants cumulatively explain only a small fraction of total heritability. Regarding the common variants, the loci identified by the current GWAS are estimated to explain only 5-10% of the genetic heritability of T2D [68]. All in a sentence, these common variants have failed to explain most of the genetic contribution to disease [69]. 

Several clinical studies assessed the predictive value of these loci for the diabetes risk. For example, a 3-year follow-up study found that the risk allele homozygotes (TT) of TCF7L2 variant rs7903146 were more likely to develop diabetes from impaired glucose tolerance than the protective allele homozygotes [70]. Two independent studies in 2008 examined genotypes of 16 and 18 T2D loci respectively, and concluded that these newly identified T2D loci provided limited predictive information of T2D beyond the clinical risk factors (e.g., family history, BMI, hepatic enzymes, smoking status which were taken into consideration [71, 72]. A series of studies performed have tried to find out the predictive and discrimitive effect of these loci on diseases risk and to identify high risk populations [57, 73]. The clinical T2D prediction models that consist of basic demographic, clinical, and laboratory predictors have C statistics ranging from 0.66 in the Rotterdam Study [74] to 0.90 in the Framingham Offspring Study [75], which were greater than the values when genotype scores alone were tested. Moreover, the addition of genotype risk scores to clinical prediction models only modestly improves the C statistic. For example, the C statistic improves from 0.903 to 0.906 with the addition of a 40-SNP score to the clinical model in the Framingham Offspring Study [74] and from 0.78 to 0.79 in participants of European ancestry from the Health Professionals Follow-up Study and Nurses’ Health Study [73] and from 0.71 to 0.73 in Han Chinese case control cohort [57]. There is one issue that should be concerned. Using genotype scores to predict T2D, it should probably be noted that many of the “clinical” risk factors which are stronger predictors of diabetes also have a genetic basis, such as obesity, smoking and family history. It could be more possible that the impact of genetics upon disease is too underestimated. Though the situation is a little bit disappointing, the future is promising. The big progress is thought to be on at least two research fronts that may improve the predictive performance of genotype information [76]. First, expanded GWAS efforts in non-European populations will allow targeted sequencing of risk loci and the identiﬁcation of true causal variants. Second, genotype information may perform better than clinical risk predictors over a longer period of the life course.

Another potential clinical implementation is in pharmacogenetics. Pharmacogenetics is the study of interactions between genetic variations and effects of drugs. However, little progression has been made on the basis of the novel identified genetic loci. In the Diabetes Prevention Program (DPP) study, the authors did not detect significant interactions between genotypes at either SNP (TCF7L2 rs7903146 and rs12255372, SLC30A8 rs13266634) and the interventions [70, 77]. However, other studies have found a significant interaction between genetic factors and drug effects. In a retrospective, observational Scottish cohort study [78], Pearson *et al.* identified that TT carriers of TCF7L2 rs12255372 variation were more likely to fail sulfonylurea treatment in a gene-dose dependent fashion; the effect of metformin response was independent of genotype. In a study focused on metformin, subjects carrying a reduced function allele in OCT-1 (organic cation transporter 1, which plays a role in hepatic metformin uptake) resulted in higher glucose levels during oral glucose tolerance test (OGTT) in metformin treated non-diabetic subjects [79]. A recent GWAS for glycemic response to metformin was performed in 1,024 Scottish individuals with T2D with replication in two cohorts including 1,783 Scottish individuals and 1,113 individuals from the UK Prospective Diabetes Study. ATM, a gene known to be involved in DNA repair and cell cycle control, was found to play a role in the effect of metformin upstream of AMP-activated protein kinase, and variation in this gene altered glycemic response to metformin [80]. A meta-analysis consisting three cohorts from Diabetes Care System West-Friesland (DCS), the Rotterdam Study and CARDS Trial, has confirmed the findings [81]. 

## GENE-ENVIRONMENTAL INTERACTION

Another consideration of post GWAS era is study of gene environmental interaction. For most complex diseases including T2D, both genetic and environmental factors are involved in the pathogenesis processes. Genetic makeup does not change, but the environmental factors are changing over the lifetime. It is very essential to study the interaction of genetic factors and environmental factors in the diseases onset, prevention procedures and intervention methods. Great progress has been seen since GWAS has reported the abundant susceptibility loci. Lifestyle and diet habit are important environmental factors. A recent meta-analysis reported that the obesogenic effect of the FTO rs9939609 minor allele was substantially diminished by physical activity [82]. The analysis comprised up to 218,166 adults and provided strong statistical evidence supporting this gene-environmental interaction. Lifestyle intervention trials generally support beneficial responses on adiposity measures regardless of FTO genotype [83, 84]. Many studies focused on dietary intake and interventions have found significant interaction with genotypes. Recently, investigators of the Cohorts for Heart and Aging Research in Genetic Epidemiology (CHARGE) consortium [85, 86] have conducted two large-scale gene-diet interaction studies. In one study [85], they included 14 cohorts to assess the interaction of 20 genetic variants known to be related to glycemic traits and zinc metabolism with dietary zinc intake (food sources) and 5 cohorts to assess the interaction with total zinc intake (food sources and supplements) on fasting glucose levels among individuals of European ancestry without diabetes. A nominally significant yet biologically plausible interaction was observed between SLC30A8 (rs11558471) and total zinc intake. Higher total zinc intake may attenuate the glucose-raising effect of the rs11558471 SLC30A8 (zinc transporter) variant. In another study [86] it was found that higher whole-grain intake was associated with a smaller reduction in fasting insulin in those with the insulin-raising allele of rs780094 (GCKR). Several reports have studied the modification effect of T2D genetic variations, IRS1 (rs2943641 [87]) and GIPR (rs2287019 [88]) on weight loss and related improvement of insulin resistance in a 2-year randomized trial: the Preventing Overweight Using Novel Dietary Strategies (POUNDS LOST) trial. The results may provide evidence for better choice of effective intervention. To use combined genetic effect (such as genetic risk score) in the gene-environmental interaction tests is a reasonable and effective way, especially when the individual genetic variation effect is minor or moderate. Qi *et al.* [90] assessed whether established genetic variants, mainly from GWAS, modify dietary patterns in predicting diabetes risk. A more Western dietary pattern significantly increased risk of T2D only among those with a high genetic risk score. Secondary analysis suggested the interaction was attributable to the red and processed meat component of the Western diet. No interaction with a prudent diet was observed. They concluded that genetic predisposition may synergistically interact with a Western dietary pattern in determining diabetes risk in men. 

## HYPERTHYROIDISM

The occurrence of Graves’ disease is related to the combined effect of genetic, environmental factors. Epidemio logical studies have confirmed that the incidence of Graves’ disease has a significant genetic predisposition [91-93]. Previous studies have identified many putative susceptibility variants for Graves’ disease. Until recently, only the major histo-compatibility complex (*MHC*) [94, 95] and cytotoxicympho-cyte antigen-4 (*CTLA-4*), *TSHR *and *PTPN22* [96-100] have been consistently found associated with Graves’ disease. Recently, the WTCCC performed a study with a genome-wide set of non-synonymous coding variants and provided evidence that three loci (*MHC, TSHR and FCRL3*) were associated with Graves’ disease in individuals of European ancestry [101]. The exploration of genome wide susceptibility loci for Graves’ disease and other thyroid diseases achieved great progression since then. So far, there are more than 20 genes were reported to be associated with thyroid volume and function, thyroid cancer and Graves’ disease (Table **2**, [102-109])]. Most of them are identified and replicated in European ancestry except that two GWAS of Graves’ Diseases were performed in Chinese and Japanese. In the Chinese study, Chu *et al.* [108] conducted a GWAS in 1,536 individuals with Graves’ disease (cases) and 1,516 controls and followed by a further replication study which included 3,994 cases and 3,510 controls. Two new susceptibility loci (the *RNA SET2-FGFR1 OP-CCR6* region at 6q27 were (Pcombined = 6.85 × 10^−10^ for rs9355610) and an intergenic region at 4p14 (Pcombined = 1.08 × 10^−13^ for rs68321 51)). The functional study showed that these newly associated SNPs were correlated with the expression levels of *RNASET2* at 6q27, of *CHRNA9* and of a previously uncharacterized gene at 4p14, respectively. Moreover, strong associations of *TSHR* and major histocompatibility complex class II variants with persistently TRAb-positive Graves’ disease were confirmed in the study.

In addition to these GWAS, some studies focused on the candidate genes in pathogenesis pathway of thyroid diseases. These studies provide more evidence of genetic basis of the diseases and may cast light on the etiology of this autoimmune disease. Graves' disease is an organ-specific autoimmune thyroid disease; the etiology of Graves’ disease may be multifactorial, but the immune response plays a central role. E-selectin, similar to L-selectin, is one of the three members of the selectin family and has been shown to mediate the recruitment of circulating leukocytes by physically supporting adhesive interactions, and participating in cell signalling and rolling. [110, 111] Furthermore, it was well documented that patients with untreated Graves’ disease had high serum levels of a soluble form of E-selectin (sE-selectin), and the concentrations of this adhesion molecule correlated with the activity of the disease, probably reflecting an ongoing immune process. [112, 113]. Chen H, *et al.* [114, 115] reported common L-selectin or E-selectin variants may be associated with susceptibility to Graves’ disease in Chinese population. Cytokines, a large group of non-enzymatic proteins, participate in the induction and effector phases of all inflammatory and immune responses, and are therefore likely to play a critical role in the development of autoimmune diseases [116]. A series of case-control studies have evaluated the associations of genetic variations of several interleukin family members with Graves’ diseases [117-125]. They reported the genetic variations in interleukin-1, 3, 4, 5, 8, 9, 12, 13, 16 and 21 were related to the Graves’ diseases in well defined Chinese case control designed studies. Another important candidate gene for thyroid diseases is the interferon-induced helicase (IFIH1) gene. IFIH1 also identified as a type 1 diabetes (T1D) susceptible loci [126] and a cause gene by re-sequencing the genomic regions initially identified by GWAS [127]. rs1990760-T was associated with decreased risk of T1D. It was found to be associated with increased risk of Graves’ disease in Caucasians [128]. *In vivo* study showed that rs1990760-T is associated with anti-dsDNA antibodies and may play a biological impact on the autoimmune disease risk allele within the interferon-( (IFN-α) pathway [129]. However, the rs1990760-T polymorphism is not related to Graves' disease in Chinese [130] or Japanese population [131]. 

## CONCLUSION

During the past several years, genetic studies of complex diseases have made substantial progression. Hundreds of susceptibility variations have been identified related to the common complex diseases and traits (T2D, obesity, hypertension, cancers, hyperthyroidism, and as well as plasma glucose levels, BMI, A1c, etc). Though the effect of most of the identified loci are moderate, often located in the intergenic or intronic regions, and small discrimination fraction from conventional clinical risk factors, the genetic findings encourage clinicians and investigators to engage much more efforts on further exploration of disease prediction, high-risk population stratification and pathogenesis study. There will be a long journey before applying the GWAS results into personalized medicine. The future studies aimed to translate the GWAS data to clinical interpretation are eagerly needed. The studies for interactions of genetic variations and environmental factors maybe a promising field to utilize the genetic variants. The successful functional and biological studies of the reported susceptibility genes depend on the identification of ‘causal’ locus, indicating rare variants to be more important.

## Figures and Tables

**Table 1.** The Susceptibility Genetic Loci for Type 2 Diabetes [by May^-20^12]. The References Listed Here Are Those That Firstly Reported the Significant Loci with P Value Less than 5 x 10^^-8^^ for the GWAS

	Year	Genes	Location	SNP	Type of SNP	Odds Ratio, 95% Confidence Interval	P-values	References
**1**	**2000**	*CAPN10*	2q37.3	9803A/G	Missense	-	-	[8] Horikawa Y, Nat Genet 2000
**2**		*PPARG*	3p25.2	rs1801282-C	Missense	1.25 [Not Reported]	0.002	[18] Altshuler D, Nat Genet 2000
**3**	**2003**	*KCNJ11*	11p15.1	rs5219-T	Missense	1. 23 [1.12–1.36] 1.14 [1.10-1.19]	1.5x10^-5^ 7x10^-11^	[19] Gloyn AL, Diabetes 2003 [28] Scott LJ, Science 2007
				rs5215-C	Missense	1.14 [1.10-1.19]	5.0x10^-11^	[22] Zeggini E, Science 2007
**4**	**2006**	*TCF7L2*	10q25.3	rs7903146-T	Intron	1.54 [Not Reported] 1.65 [1.28-2.02] 1.38 [Not Reported]	2.1x10^-9^ 2.0x10^-34^ 2x10^-10^	[12] Grant SF, Nat Genet. 2006; [24] Sladek R, Nature 2007 [29] Steinthorsdottir V, Nat Genet 2007
			10q25.2	rs7901695-C	Intron	1.37 [1.31-1.43]	1.0x10^-48^	[22] Zeggini E, Science 2007
			10q25.2	rs4506565-T	Intron	1.36 [1.20-1.54]	5x10^-12^	[23] WTCCC, Nature 2007
**5**	**2007**	*SLC30A8*	8q24.11	rs13266634-C	cds-synon	1.18 [0.69-1.67] 1.12 [1.07-1.16]	6x10^-8^ 5x10^-8^	[24] Sladek R, Nature 2007 [22] Zeggini E, Science 2007
**6**		*WFS1*	4p16.1	rs10010131-T	Intron	0.90 [0.86-0.93]	1.4x10^-7^	[25] Sandhu MS, Nat Genet. 2007
				rs6446482-C	intron	0.90 [0.87-0.94]	3.4x10^-7^	[25] Sandhu MS, Nat Genet 2007
				rs1801214-T	cds-synon	1.13 [1.08-1.18]	3x10^-8^	[34] Voight BF, Nat Genet 2010
**7**		*TCF2 (HNF1B)*	17q12	rs7501939-C	intron	0.91 [0.87–0.94]	9.2x10^-7^	[26] Gudmundsson J, Nat Genet 2007
			17q12	rs4430796-A	intron	0.91 [0.87–0.94]	2.7x10^-7^	[26] Gudmundsson J, Nat Genet 2007
**8**		*HHEX*	10q23.33	rs1111875-C	intergenic	1.13 [1.08-1.17]	6x10^-10^	[28] Scott LJ, Science 2007
				rs5015480-C	Intergenic	1.18 [1.13-1.23]	1x10^-15^	[34] Voight BF, Nat Genet 2010
**9**		*IGF2BP2*	3q27.2	rs4402960-T	Intron	1.14 [1.11-1.18]	9x10^-16^	[22] Zeggini E, Science 2007
				rs6769511-C	Intron	1.23 [1.15-1.31]	1x10^-9^	[27] Unoki H, Nat Genet 2008
**10**		*FTO*	16q12.2	rs8050136-A	Intron	1.23 [1.18-1.32]	9x10^-16^	[22] Zeggini E, Science 2007
				rs9939609-A	Intron	1.34 [1.17-1.52]	2x10^-7^	[23] WTCCC, Nature 2007
**11**		*CDKAL1*	6p22.3	rs10946398-C	Intron	1.16 [1.10-1.22]	1x10^-8^	[22] Zeggini E, Science 2007
				rs7754840-C	Intron	1.12 [1.08-1.16]	4x10^-11^	[28] Scott LJ, Science 2007
				rs7756992-G	Intron	1.2 [1.13-1.27]	8x10^-9^	[29] Steinthorsdottir V, Nat Genet 2007
				rs9465871-C	Intron	1.18 [1.04-1.34]	3x10^-7^	[23] WTCCC, Nature 2007
				rs4712524-G	intron	1.22 [1.15-1.31]	3x10^-10^	[27] Unoki H, Nat Genet 2008
**12**		*CDKN2A, CDKN2B*	9p21.3	Rs564398-T	Intron	1.13 [1.08-1.19]	1x10^-8^	[22] Zeggini E, Science 2007
				rs10811661-T	Intergenic	1.2 [1.14-1.25]	8x10^-15^	[28] Scott LJ, Science 2007
				rs2383208-A	Intergenic	1.34 [1.27-1.41]	2x10^-29^	[30] Takeuchi F, Diabetes 2009
				rs7018475-?	intergenic	1.35 [1.18-1.56]	3x10^-8^	[45] Huang J, Eur J Hum Genet 2012
**13**	**2008**	*JAZF1*	7p15.1	rs864745-T	Intron	1.1 [1.07-1.13]	5x10^-14^	[31] Zeggini E, Nat Genet 2008
**14**		*CDC123 - CAMK1D*	10p13	rs12779790-G	Intergenic	1.11 [1.07-1.14]	1x10^-10^	[31] Zeggini E, Nat Genet 2008
**15**		*TSPAN8 - LGR5*	12q21.1	rs7961581-C	Intergenic	1.09 [1.06-1.12]	1x10^-9^	[31] Zeggini E, Nat Genet 2008
**16**		*THADA*	2p21	rs7578597-T	Mssense	1.15 [1.10-1.20]	1x10^-9^	[31] Zeggini E, Nat Genet 2008
**17**		*ADAMTS9 - MAGI1*	3p14.1	rs4607103-C	Intergenic	1.09 [1.06-1.12]	1x10^-8^	[31] Zeggini E, Nat Genet 2008
**18**		*NOTCH2*	1p12	rs10923931-T	Intron	1.13 [1.08-1.17]	4x10^-8^	[31] Zeggini E, Nat Genet 2008
**19**		*KCNQ1*	11p15.4	rs2237892-C	Intron	1.4 [1.34-1.47]	2x10^-42^	[33] Yasuda K, Nat Genet 2008
				rs2237897-C	intron	1.33 [1.24-1.41]	1x10^-16^	[27] Unoki H, Nat Genet 2008
				rs231362-G	Intron	1.08 [1.06-1.10]	3x10-13	[34] Voight BF, Nat Genet 2010
				rs2237895-C	Intron	1.29 [1.19-1.40]	1x10^-9^	[38] Tsai FJ, PLoS Genet 2010
**20**	**2009**	*LOC64673, IRS1*	2q36.3	rs2943641-C	Intergenic	1.19 [1.13-1.25]	9x10^-12^	[35] Rung J, Nat Genet 2009
**21**	**2010**	*RBMS1, ITGB6*	2q24.2	rs7593730-C	Intron	1.11 [1.08-1.16]	4x10^-8^	[36] Qi L, Hum Mol Genet 2010
**22**		*CENTD2*	11q13.4	rs1552224-A	intron	1.14 [1.11-1.17]	1x10^-22^	[34] Voight BF, Nat Genet 2010
**23**		*KIAA1486 - IRS1(IRS1)*	2q36.3	rs7578326-A	intergenic	1.11 [1.08-1.13]	5x10^-20^	[34] Voight BF, Nat Genet 2010
**24**		*BCL11A*	2p16.1	rs243021-A	intergenic	1.08 [1.06-1.10]	3x10^-15^	[34] Voight BF, Nat Genet 2010
**25**		*MTNR1B*	11q14.3	rs1387153-T	intergenic	1.09 [1.06-1.11]	8x10^-15^	[34] Voight BF, Nat Genet 2010
**26**		*ZBED3*	5q13.3	rs4457053-G	intergenic	1.08 [1.06-1.11]	3x10^-12^	[34] Voight BF, Nat Genet 2010
**27**		*PRC1*	15q26.1	rs8042680-A	intron	1.07 [1.05-1.09]	2x10^-10^	[34] Voight BF, Nat Genet 2010
**28**		*KLF14*	7q32.3	rs972283-G	intergenic	1.07 [1.05-1.10]	2x10^-10^	[34] Voight BF, Nat Genet 2010
**29**		*DUSP9*	Xq28	rs5945326-A	intergenic	1.27 [1.18-1.37]	3x10^-10^	[34] Voight BF, Nat Genet 2010
**30**		*TP53INP1*	8q22.1	rs896854-T	intron	1.06 [1.04-1.09]	1x10^-9^	[34] Voight BF, Nat Genet 2010
**31**		*ZFAND6*	15q25.1	rs11634397-G	intergenic	1.06 [1.04-1.08]	2x10^-9^	[34] Voight BF, Nat Genet 2010
**32**		*HMGA2*	12q14.3	rs1531343-C	UTR-3	1.1 [1.07-1.14]	4x10^-9^	[34] Voight BF, Nat Genet 2010
**33**		*HNF1A*	12q24.31	rs7957197-T	intron	1.07 [1.05-1.10]	2x10^-8^	[34] Voight BF, Nat Genet 2010
**34**		*C2CD4A,C2CD4B*	15q22.2	rs7172432-A	intergenic	1.11 [1.08-1.14]	9x10^-14^	[37] Yamauchi T, Nat Genet 2010
**35**		*PTPRD*	9p24.1	rs17584499-T	intron	1.57 [1.36-1.82]	9x10^-10^	[38] Tsai FJ, PLoS Genet 2010
**36**		*SRR*	17p13.3	rs391300-G	intron	1.28 [1.18-1.39]	3x10^-9^	[38] Tsai FJ, PLoS Genet 2010
**37**		*CDC123,CAMK1D*	10p13	rs10906115-A	intergenic	1.13 [1.08-1.18]	1x10^-8^	[39] Shu XO, PLoS Genet 2010
**38**		*SPRY2*	13q31.1	rs1359790-G	intergenic	1.15 [1.10-1.20]	6x10^-9^	[39] Shu XO, PLoS Genet 2010
**39**	**2011**	*C6orf57*	6q13	rs1048886-G	missense	1.54 [1.32-1.80]	3x10^-8^	[40] Sim X, PLoS Genet. 2011
**40**		*AP3S2*	15q26.1	rs2028299-C	UTR-3	1.1 [1.07-1.13]	2x10^-11^	[41] Kooner JS, Nat Genet 2011
**41**		*HMG20A*	15q24.3	rs7178572-G	intron	1.09 [1.06-1.12]	7x10^-11^	[41] Kooner JS, Nat Genet 2011
**42**		*GRB14*	2q24.3	rs3923113-A	intergenic	1.09 [1.06-1.13]	1x10^-8^	[41] Kooner JS, Nat Genet 2011
**43**		*ST6GAL1*	3q27.3	rs16861329-G	intron	1.09 [1.06-1.12]	3x10^-8^	[41] Kooner JS, Nat Genet 2011
**44**		*VPS26A*	10q22.1	rs1802295-A	UTR-3	1.08 [1.05-1.12]	4x10^-8^	[41] Kooner JS, Nat Genet 2011
**45**		*MAEA*	4p16.3	rs6815464-C	Intron	1.13 [1.10-1.16]	2x10^-20^	[42] Cho YS, Nat Genet 2011
**46**		*GLIS3*	9p24.2	rs7041847-A	Intron	1.1 [1.07-1.13]	2x10^-14^	[42] Cho YS, Nat Genet 2011
**47**		*FITM2,R3HDML,HNF4A*	20q13.12	rs6017317-G	Intergenic	1.09 [1.07-1.12]	1x10^-11^	[42] Cho YS, Nat Genet 2011
**48**		*GCC1,PAX4*	7q32.1	rs6467136-G	intergenic	1.11 [1.07-1.14]	5x10^-11^	[42] Cho YS, Nat Genet 2011
**49**		*PSMD6*	3p14.1	rs831571-C	Intergenic	1.09 [1.06-1.12]	8x10^-11^	[42] Cho YS, Nat Genet 2011
**50**		*ZFAND3*	6p21.2	rs9470794-C	Intron	1.12 [1.08-1.16]	2x10^-10^	[42] Cho YS, Nat Genet 2011
**51**		*PEPD*	19q13.11	rs3786897-A	Intron	1.1 [1.07-1.14]	1x10^-8^	[42] Cho YS, Nat Genet 2011
**52**		*KCNK16*	6p21.2	rs1535500-T	intron	1.08 [1.05-1.11]	2x10^-8^	[42] Cho YS, Nat Genet 2011
**53**	**2012**	*RBM43, RND3*	2q23.3	rs7560163-C	Intergenic	1.33 [1.19-1.49]	7x10^-9^	[43] Palmer ND, PLoS One 2012
**54**		*ANK1*	8p11.21	rs515071-C	intron	1.18 [1.12-1.25]	1x10^-8^	[44] Imamura M, Hum Mol Genet 2012

**Table 2.** The Susceptibility Genetic Loci for Thyroid Diseases [by May^-20^12]

Disease/Trait	Gene(s)	Location	Strongest SNP-Risk Allele	Initial/Replication Sample	Type of SNP	P-Value	OR or beta	95% Confidence Interval	References
**Thyroid function**	PDE8B	5q13.3	rs2046045-T	European/ European	Intron	2.79x10^-27^	-0.115	[0.093-0.137] Unit decrease	[102] Rawal R, Hum Mol Genet. 2012
	CAP2B	1p36	rs10917477-A		Intergenic	1.54x10^-8^	-0.058	[0.038-0.078] Unit decrease	
	LOC440389	16q23	rs3813582-T		Intergenic	5.63x10^-10^	0.068	[0.046-0.090] Unit increase	
	NR3C2	4q31	rs10028213-C		Intergenic	2.88x10^-10^	0.084	[0.059-0.109] Unit increase	
**Thyroid cancer**	MBIP	14q13.3	rs116909374-T	European/ European	Intergenic	5x10^-11^	2.09	[1.68-2.60]	[103] Gudmundsson J, Nat Genet 2012
	NRG1	8p12	rs2439302-G		Intron	2x10^-9^	1.36	[1.23-1.50]	
	DIRC3	2q35	rs966423-C			1x10^-9^	1.34	[1.22-1.47]	
	FOXE1	9q22.33	rs965513-A		Intergenic	2x10^-27^	1.75	[1.59-1.94]	[104] Gudmundsson J, Nat Genet 2009
	NKX2-1	14q13.3	rs944289-T		Intergenic	2x10^-9^	1.37	[1.24-1.52]	
**Thyroid volume**	CAPZB	1p36.13	rs12045440-T	European/ European	Intergenic	2x10^-11^	1.38	[1.26-1.51]	[105] Teumer A, Am J Hum Genet 2011
	CAPZB	1p36.13	rs12138950-A		Intergenic	3x10^-18^	0.1	[0.08-0.12] Unit decrease	
	MAF	16q23.2	rs3813579-A		Intergenic	4x10^-10^	1.32	[1.21-1.44]	
	MAF	16q23.2	rs17767419-T		Intergenic	9x10^-15^	0.07	[0.05-0.09] Unit increase	
	CAPZB	1p36.13	rs10917468-C		Intergenic	1x10^-14^	1.52	[1.37-1.69]	
	C15orf33, FGF7	15q21.2	rs4338740-C		Intron; Intron	3x10^-13^	1.45	[1.32-1.59]	
	C15orf33, FGF7	15q21.2	rs4338740-T		Intron; Intron	1x10^-12^	0.07	[0.05-0.09] Unit decrease	
**Thyroid Stimulating Hormone**	HACE1	6q16.3	rs9322817-?	Framingham/NR	Intron	7x10^-6^	NR	NR	[106] Hwang SJ, BMC Med Genet 2007
	RAPGEF5	7p15.3	rs10499559-?		Intergenic	8x10^-6^	NR	NR	
	Intergenic	7p21.1	rs6977660-?		Intron	4x10^-6^	NR	NR	
**Hypothyroidism**	FOXE1	9q22.33	rs7850258-?	European/ European	Intergenic	4x10^-9^	1.23	[1.04-1.47]	[107] Denny JC, Am J Hum Genet 2011
**Graves' Disease**	HLA, DPB1	6p21.32	rs2281388-T	Chinese/ Chinese	Intergenic	2x10^-65^	1.64	[1.55-1.74]	[108] Chu X, Nat Genet 2011
	HLA-B	6p21.33	rs1521-T		Intergenic	2x10^-65^	1.92	[1.78-2.08]	
	MUC21, C6orf15	6p21.33	rs4947296-C		Intergenic	4x10^-51^	1.77	[1.65-1.91]	
	HLA, DRB1, DQA1, DQB1	14q31.1	rs6457617-T		Intron	7x10^-33^	1.4	[1.32-1.48]	
	TSHR	2q33.2	rs12101261-T		Intergenic	7x10^-24^	1.35	[1.28-1.43]	
	CD28, CTLA4	4p14	rs1024161-T		Intergenic	2x10^-17^	1.3	[1.23-1.38]	
	RHOH, CHRNA9	1q23.1	rs6832151-G		Intron	1x10^-13^	1.24	[1.17-1.31]	
	FCRL3	6q27	rs3761959-A		Intergenic	2x10^-13^	1.23	[1.17-1.30]	
	RNASET2, FGFR1OP	6q15	rs9355610-G		Intron	7x10^-10^	1.19	[1.13-1.26]	
	BACH2, MAP3K7	6p21.32	rs370409-T		Intron	2x10^-6^	1.15	[1.09-1.22]	
	ABO	9q34.2	rs505922-T			8x10^-6^	1.13	[1.07-1.20]	
	MHC	6p21.32	rs2273017-A	Japanese/ Japanese	Intron	2x10^-22^	1.53	[1.40-1.66]	[109] Nakabayashi K, J Hum Genet 2011
	MHC	6p22.1	rs3893464-G		Intergenic	2x10^-20^	1.53	[1.39-1.67]	
	MHC	6p22.1	rs4313034-T		Neargene-5	2x10^-15^	1.67	[1.47-1.90]	
	MHC	6p21.33	rs3132613-C		Intergenic	1x10^-13^	1.43	[1.30-1.57]	
	MHC	6p21.33	rs4248154-C		Intron	1x10^-13^	1.38	[1.27-1.50]	
	MHC	6p21.31	rs4713693-T			7x10^-13^	1.4	[1.28-1.53]	
	MHC	6p21.31	rs9394159-T			4x10^-12^	1.36	[1.24-1.48]	
